# Client experiences with “Dynamic Choice Prevention,” a model for flexible patient‐centred HIV prevention delivery in rural Eastern Africa

**DOI:** 10.1002/jia2.26336

**Published:** 2024-07-17

**Authors:** Carol S. Camlin, Titus Arunga, Jason Johnson‐Peretz, Cecilia Akatukwasa, Fredrick Atwine, Angeline Onyango, Lawrence Owino, Moses R. Kamya, Maya L. Petersen, Gabriel Chamie, Elijah Kakande, Jane Kabami, Laura B. Balzer, Diane V. Havlir, James Ayieko

**Affiliations:** ^1^ University of California, San Francisco (UCSF), Obstetrics, Gynecology & Reproductive Sciences San Francisco California USA; ^2^ Kenya Medical Research Institute (KEMRI) Kisumu Kenya; ^3^ Infectious Diseases Research Collaboration Kampala Uganda; ^4^ Department of Medicine Makerere University College of Health Sciences Kampala Uganda; ^5^ University of California, Berkeley, Biostatistics, Epidemiology, and Computational Precision Health Berkeley California USA; ^6^ University of California, San Francisco (UCSF), Medicine San Francisco California USA

**Keywords:** pre‐exposure prophylaxis, post‐exposure prophylaxis, HIV self‐testing, differentiated care, HIV stigma, sub‐Saharan Africa

## Abstract

**Introduction:**

Identifying the optimal approaches to offering HIV prevention to meet the needs of those at risk is a high priority, particularly given the expanding toolkit of biomedical HIV prevention options. An ongoing study in rural East African communities evaluated the uptake of choices in product, testing mode and location of care delivery through a structured patient‐centred HIV prevention delivery model. In this qualitative study, we sought to understand clients’ experiences of this “dynamic choice prevention model” (DCP) and highlight pathways of action to inform HIV prevention delivery models.

**Methods:**

In‐depth semi‐structured interviews were conducted from November 2021 through March 2022 with a purposively selected sample of *n* = 56 participants in DCP trials (across outpatient departments, antenatal clinics and community settings), and *n* = 21 healthcare providers (total *n* = 77). A seven‐person multi‐regional team translated and inductively coded transcript data. We used a framework analysis approach to identify emergent themes.

**Results:**

Individuals taking up HIV pre‐exposure prophylaxis (PrEP) reported feelings of relief, liberation from fears of acquiring HIV and satisfaction with being able to take action despite partners’ behaviours. Couples used a range of approaches afforded by the study to persuade partners to get tested and opt for PrEP. Post‐exposure prophylaxis (PEP) use was less common, although women welcomed it in the event of sexual coercion or assault. Participants discussed switching from PEP to PrEP after familiarizing themselves with usage and ascertaining ongoing risk. Participants felt respected by providers, trusted them and appreciated being able to contact them directly for telephone support. Prevention uptake was hindered by stigma, limited experience with and knowledge of prevention methods, gendered and generational power dynamics within intimate partnerships and families, and negative perceptions of methods due to the products themselves. Participants anticipated long‐acting injectable PrEP could solve their challenges regarding pill size, daily pill burden and the likelihood of unwanted disclosure.

**Conclusions:**

Diverse preferences and barriers to uptake of prevention require a choice of HIV prevention options, locations and delivery modalities—but in addition, flexible, competent and friendly care provision is crucial to promote uptake. Helping clients feel valued, and addressing their unique needs and challenges, enables their agency to prioritize their health.

## 1 INTRODUCTION

Offering individuals a choice of HIV prevention options is a key element of patient‐centred care, and crucial for reaching HIV prevention goals [1, 2, 3]. Biomedical tools to prevent HIV acquisition beyond oral pre‐ and post‐exposure prophylaxis (PrEP and PEP) include long‐acting injectables (e.g. cabotegravir) [4, 5] and intra‐vaginal silicone rings [6, 7]. HIV self‐testing has increased options for individuals to know their HIV status and test regularly, expanding the prevention toolkit [8]. Several studies conducted multiple discrete choice experiments to identify clients’ preferences for prevention methods and delivery approaches, but few examine clients’ desire for choice *per se*, although literature shows diverse preferences within and across populations [9, 10]. Identifying optimal approaches within this range for at‐risk individuals is a high priority given the expanding toolkit [2, 3].

The Sustainable East Africa Research in Community Health (SEARCH) Consortium conducted three studies (NCT04810650) to test whether a patient‐centred dynamic choice model for HIV prevention delivery (DCP) would lead to higher uptake of biomedical prevention. In intervention arms, patients had choice of product, HIV testing mode and site of care delivery [11]. This qualitative study, embedded within SEARCH, sought to deepen understanding of how and when people take up various prevention methods, why they stop or switch and what addressable barriers to prevention uptake may exist. We highlight the interaction of DCP intervention elements in an open‐ended pathway with potential feedback loops as participants explored different testing modality choices, service delivery locations and products, providing evidence for the intervention's mechanism(s) of action. We define “pathways” as longer causal chains, and “meaningful mechanism” as the set of interacting components of catalysts, supports, meanings and individual pathways within an interrelated whole [12, 13, 14].

## 2 METHODS

### 2.1 Study context

This cross‐sectional qualitative study was conducted in rural communities in Uganda and Kenya participating in the SEARCH DCP trials. DCP was offered in antenatal clinics (ANCs), outpatient departments (OPDs) and community settings via community health workers (CHWs). Components included training providers on offering prevention product choice responsive to clients’ desires, including self‐ or clinician‐administered HIV testing, clinic or offsite visits and choice of products. All clients received mobile phone access to clinicians (24/7), integrated reproductive health services, and an assessment of uptake and adherence barriers with personalized plans to address challenges. The DCP trial testing facility‐based versus clinician‐supported CHW‐delivered services was found to increase biomedical prevention coverage by 27.5% among adult men and women at risk of HIV acquisition [15]. The trial testing DCP delivery among women seen at ante/postnatal care clinics increased prevention coverage by 40% [16].

### 2.2 Sampling

We conducted in‐depth semi‐structured interviews (IDIs) with a purposively selected sample of 77 SEARCH participants (37 in Uganda and 40 in Kenya) and 21 providers who delivered DCP services (11 in Uganda and 10 in Kenya). The sample was balanced by region, gender and cohort (with 19 from ANC clinics, 19 from the OPD cohort and 18 from the CHW cohort) and for providers, by cadre (clinical officers, nurses and CHWs).

### 2.3 Data collection

From November 2021 through March 2022, a gender‐balanced team of trained qualitative researchers conducted audio‐recorded IDIs in English, DhoLuo (AO, LO, TOA) and Runyankole (CA, FA) in private locations preferred by participants. IDIs explored participants’ experiences with the intervention; prevention methods preference, experiences and reasons for switching; provider interactions; community prevention methods perceptions; and peer discussions about HIV, prevention and stigma. Interview guides were informed by theories of gender [17, 18] and stigma [19, 20], and where appropriate, drew upon on constructs from theories of health and social behaviour including perceived risk, perceived severity [21], method efficacy beliefs, subjective norms [22], and self‐ and vicarious efficacy [23]. Provider IDIs centred on providing DCP services and perceptions of clients’ needs and preferences. Interviewers transcribed and translated recordings into English.

Additionally, at weeks 24 and 48, the SEARCH DCP study administered surveys to all participants on method use during the previous months (PrEP, PEP, condoms only, none), location choice (clinic vs. community) and HIV testing type (rapid vs. HIV self‐test). We used these data to generate alluvial graphs summarizing choices over time among the qualitative study participants at baseline, week 24 and week 48 (Figure 2).

### 2.4 Analysis

We [JJP and CSC] developed an *a priori* codebook using concepts from the interview guides, then the full qualitative team [LO, AO, CA, FA, TOA, JJP, CSC] generated inductive codes using a team‐based approach to Charmaz's two‐stage method: multiple team members conducted open line‐by‐line coding of an initial set of transcripts, discussed and developed potential focused codes [24, 25], then integrated and organized these into a final codebook with definitions and usage guidelines. Codes were applied using Dedoose software with each transcript coded by one team member; questions about difficult‐to‐code segments were discussed with the full team to achieve consensus. The team then used a framework analysis approach [26, 27] to organize coded data, identify emergent themes and summarize findings.

#### 2.4.1 Position statement

Five team authors who collected and analysed data have a first‐hand understanding of the participants’ lived social contexts [FA, CA, LO, AO, TOA]. Their local expertise ensured data interpretation captured the nuances of participant understandings and cultural contexts.

### 2.5 Ethical approval

All interview participants provided written informed consent. The University of California San Francisco Committee on Human Research, the Makerere University School of Medicine Research and Ethics Committee, the Uganda National Council of Science and Technology, and the Ethical Review Committee of the Kenya Medical Research Institute approved this study.

## 3 RESULTS

**Figure 1:**
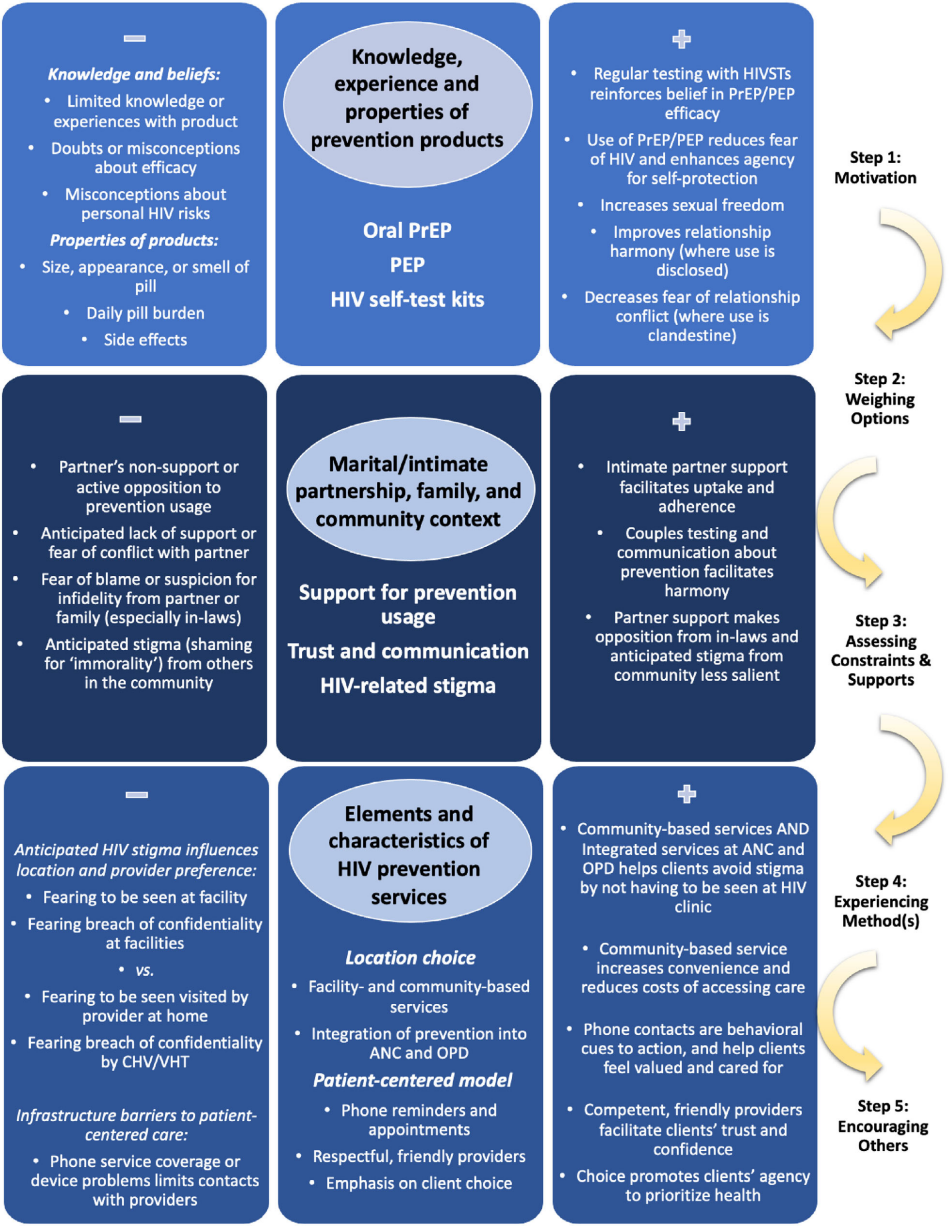
Factors contributing to HIV prevention uptake and countervailing forces. ANC, antenatal care clinic; CHV/VHT, community health volunteer/village health team; HIVST, HIV self‐test; OPD, outpatient department; PEP, HIV post‐exposure prophylaxis; PrEP, HIV pre‐exposure prophylaxis.

We describe evidence for the intervention's mechanism of action related to prevention options (PrEP, PEP and HIV testing options) and location type (facility or community‐based, including home‐based care), then present findings countervailing these pathways of action. We note steps within observed uptake pathways for each method, positioning these steps within a generalized meaningful mechanism for uptake. Figure 1 displays the factors contributing to steps in this pathway, along with granular components in the uptake of prevention across three main domains (products, social context and services). Positive and negative elements influence individuals’ uptake of these methods within each domain and progression along the steps.

**Figure 2:**
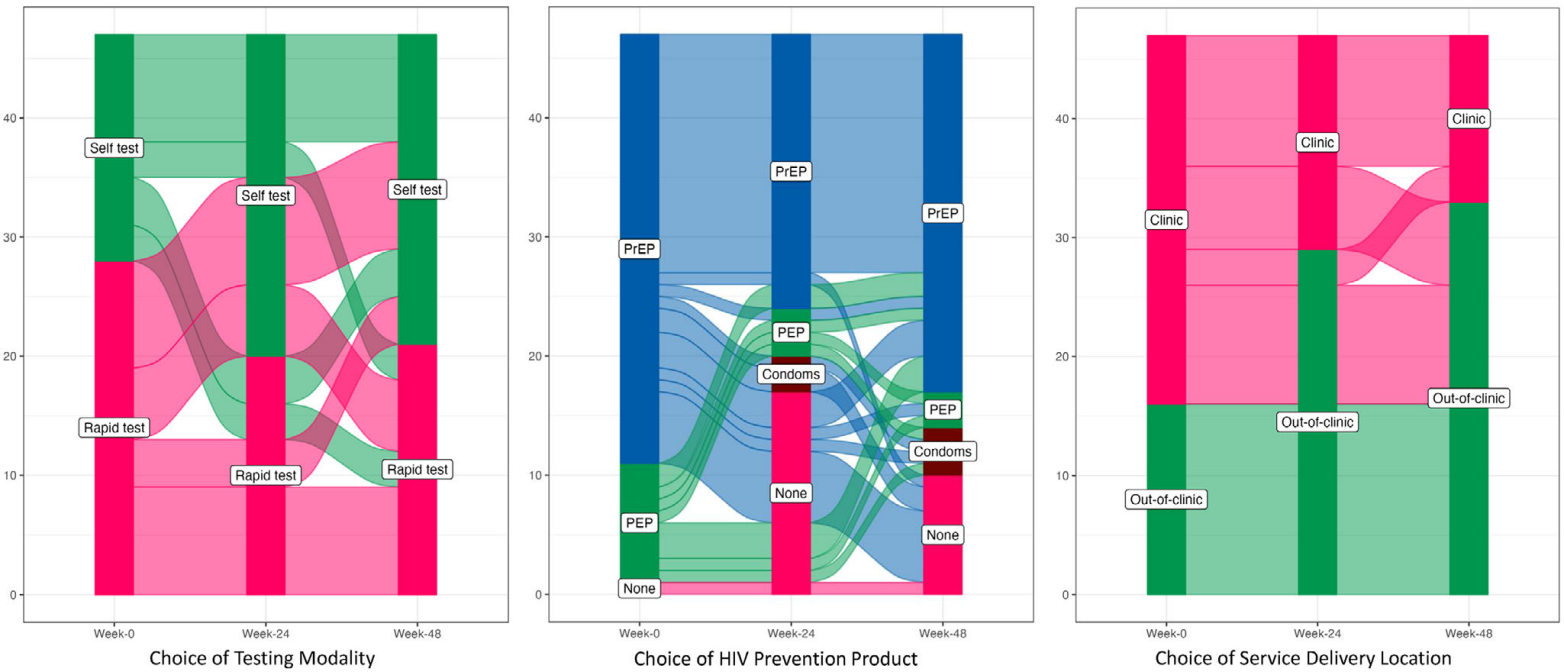
Changes in choice of testing modality, HIV prevention product and service delivery location over time.

Figure 2 shows three alluvial graphs of changes in choice (testing modality, HIV prevention method and service delivery location) for the *n* = 56 DCP client study participants. Clients’ preference for out‐of‐clinic, CHW‐delivered community‐based services increased from weeks 0, 24 to 48. More clients using facility‐based HIV testing switched to HIV self‐testing over the period compared to the reverse. Prevention method use switching was more varied across the three time points in this qualitative sub‐sample of trial participants.

### 3.1 Positive experiences and potential pathways of intervention action

#### 3.1.1 Prevention options

***Oral PrEP***. Multiple patients expressed satisfaction with PrEP as their preferred method. Successful PrEP use resulted in feelings of agency in protecting oneself from HIV acquisition, regardless of a partner's behaviour:

“…I was very happy at heart because we have been ‘walking on broken glass’. It's not that one knows they will meet so‐and‐so where they are going—[you] end up being intimate, yet you don't have any idea about their health status. I felt so relieved because sometimes you really want to be intimate with someone, but the fear is great …” *32 y.o. male, Uganda (OPD)*

PrEP allowed participants to address what may have felt like an inevitable risk of HIV, highlighting the initial step on the pathway of action: motivation to try a new prevention method followed by evaluation of options (a second step). Two young people expressed satisfaction with PrEP in comparison with condoms:

“…Well, I think PrEP was the best because when you are using it, you are protected all the time…When you are using condoms, you don't enjoy it. […] Therefore, if you cannot abstain – and I am very sure our current generation, we cannot abstain – PrEP will help you if you cannot use condom; you are safe…” *18 y.o. male, Kenya (OPD)*

“…I feel medications are the best options because men have got funny, weird characters; someone may tell you that he has put on condom, but […] he had made a hole or removed it. You may think you are safe, yet you had unsafe sex. Medications on the other hand—you are very sure when you are taking them you are safe; you cannot be worried…” *18 y.o. female, Kenya (ANC)*

Interpersonal dynamics made condom use an unreliable prevention method, while PrEP permitted self‐responsibility. Participants candidly expressed how assessing the social constraints to taking up methods affected their choices to move forward with any given option, indicating a third step on the pathway. PrEP use was often, though not always, supported by partners who recognized their own behaviour could put a partner at risk, allowing them to avoid potential disputes over whether to test as a couple. Positive experiences with method use, a fourth step, also led individuals to encourage friends to take up PrEP, forming a fifth component of the pathway. One man described having helped a female friend start taking PrEP:

“…Her husband is cheating on her with other women… I advised her to go to the doctor to give her drugs which prevent HIV. I encouraged her that once she is using PrEP, she cannot be infected with the virus if she is adhering well to her medication. That is how I managed to convince her to come for these services.” *22 y.o. male, Kenya (OPD)*

Having an HIV prevention option that was going to work despite what sexual partners chose to do conferred a sense of self‐efficacy, which in turn led individuals to share their positive experiences with others.

Thus, our participants’ narratives suggest that the PrEP uptake pathway follows five general steps, beginning with a self‐risk assessment and motive, which then prompts the participant to evaluate options. A pivotal third step is to assess the social constraints and social space for moving forward with PrEP. The fourth step is to try the method, and the fifth step comes when participants share their experience with friends—potentially widening the social space for subsequent methods uptake. This pattern was repeated with subtle variations for other methods.

***PEP***. In settings where individuals felt non‐consensual sex is common, or where individuals feared using PrEP could elicit violence from their partner, PEP was an essential prevention tool: “…[PEP] is well because at times you may be forced into sex. So when you rush to the doctors then help can be offered.” *43 y.o. female, Uganda (OPD)* Overall, participants reported fewer experiences with PEP, partly because some were told about PEP only after asking their providers questions about HIV prevention, and others not at all.

“…They did not tell me about PEP, they only told me about PrEP. They told me about PEP when I came during the last visit. I asked them if I could carry it home and they said I cannot […]. It is used when you have sex with someone you suspect to be HIV positive, and you should come to the facility and get it within 72 hours of exposure… Personally, I wished to have them with me…” *38 y.o. female, Kenya (OPD)*

Notably, some interviews were conducted early during the intervention, before providers were re‐trained to improve intervention fidelity and encourage clients to take a “pill in pocket” (i.e. carry PEP home in case of emergencies). Further, some participants confused PEP and PrEP. Unable to fully evaluate options due to lack of knowledge, the pathway to PEP was foreshortened for these participants.

***HIV self‐testing***. Several participants expressed relief about being able to track their serostatus more frequently using HIV self‐tests. Once shown how to use the test, participants found self‐testing easy:

“…It was easy for me because I have a friend who tests from the anti‐retroviral therapy (ART) clinic and she was always given this equipment so she taught me how to use it [. . .] She one time gave me the equipment and I tested three people to know if I can do this…” *39 y.o female, Uganda (OPD)*

In addition to having friends to show them how to test, participants mentioned testing their sexual partners or themselves after a potentially risky encounter: “…the benefit of partner testing is that you would be able to know your partners’ HIV status and in case they are infected—then the uninfected person would be able to understand and know how to take care.” *15 y.o. female, Kenya (VHT)*

Participants recognized that self‐testing allowed them to take precautions with their partners or reassure themselves that PEP is effective. In this sense, participants liked the combination of PEP plus a self‐test:

“…I don't know the HIV status of all the women I relate with. So what I do always, after sleeping with them, I get back home, think of myself having contracted HIV virus, I take my medicine [PEP], and after some time when I carry out the HIV test, I find myself safe…” *22 y.o. male, Uganda (VHT)*

The above accounts highlight the importance of HIV self‐testing for many on the pathway to PEP/PrEP uptake: while some used testing to ascertain partners’ status to decide whether or not to take up a method, others adopted a method then used self‐testing as a way to see whether it “worked,” before discussing it with others. This not only created another potential feedback loop, but altered the field in which the intervention played out by giving participants a means of monitoring their status themselves.

#### 3.1.2 Mode of delivery

***Service location***. Participants appreciated having a choice about the options of home visits (including via CHWs), clinic appointments (at not only HIV clinic but facility outpatient and ANCs) and phone check‐ins. **Home visits** reduced the financial outlay for travel, and reduced the risk of going to the clinic only to find that providers had already left for the day. Home visits helped participants stay adherent by getting their medication refills on time, and also provided an opportunity for education and lengthier in‐person discussions.

“…I feel happy always because when they come home, already I know what they want … If they find me with other people, I will not hide because I am not sick; instead, I am trying to protect my life. Therefore, I do it openly so that in case my visitor can develop an interest in the study then let him or her join.” *42 y.o. female, Kenya (ANC)*

In this respect, the mode of delivery allowed participants to hop over step three (assessing constraints and one's ability to overcome them) and immediately engage in step five (telling others about the method).

***Community health workers***. Many participants felt receiving services from a CHW (sometimes referred to as a “CHV” or community health volunteer) was convenient. CHWs generally were said to be trusted members of the community, and accessible to clients who lived nearby.

“I have a relative who serves as a Community Health Volunteer in our village; she further encouraged her [my first wife] to use the drugs because of my inherited [second] wife. I love that CHV because she is a very free mother, and she usually visits my home just to talk about health matters.” *39 y.o. male, Kenya (CHW)*

They served community members by delivering medication to them, and also educated those around them. This increased trust in their credibility and promoted prevention uptake by motivating people to evaluate prevention options (step two), sometimes including convincing spouses to permit their partners to take PrEP.

***Facility‐based care***. Some participants specifically preferred a hospital location rather than CHW or home visits, since going to the hospital protected them from stigma and nosey neighbours, a feature of assessing social constraints (step three):

“…I think hospital and center are the best because when I come to the hospital, no one will know the reason why I have come […] When you come to the center, you will advertise some other health conditions and the prevention service will just be among the services…” *32 y.o. female, Kenya (ANC)*

***The antenatal clinic*** acted as a resource for educating women about HIV prevention options with sensitivity to difficult marital situations, while shielding new mothers from the threat of others assuming they are HIV positive:

“We … were told that when we are done with antenatal care and are interested in the [HIV prevention] service, we could reach out to them. … What would I be doing in the HIV clinic if I am not sick? I was lucky when I came for antenatal care…” *36 y.o. female, Uganda (ANC)*

***Phone check‐ins*** acted as behavioural cues to alert clients to adhere to medication and keep appointments. They helped clients feel valued:

“…She said, ‘I am a nurse, and you were to come for your medication; unfortunately, you did not – what could be the problem?’ Then I replied, ‘I forgot, but I would come the following day.’ I set them free to remind me about my appointment date because I may forget… it was good because those medications are helpful to me. When I remain with around two pills and they remind me about my scheduled date, I feel good…” *33 y.o. female, Kenya (ANC)*

This contact created a positive feedback loop where patients felt themselves worthy of self‐care, leading to greater self‐efficacy for adherence. In this respect, phone check‐ins supported both step four—personal experience with method use—and step one, being motivated towards self‐care. These benefits also extended to CHWs, who linked community members to health services. As one commented, “clients gain morale when you call them and also can't fear you, so they easily tell you what they want” (*41 y.o. male, Uganda*). Phone calls were useful for both clients and providers, and allowed clients to raise concerns not related to HIV:

“The participants sometimes call me, especially those who get complications for example headaches and stomachaches, saying they need to meet […] to get medication. I call the participants because I always need to know how they are progressing…” *42 y.o. female, Uganda*

Confidentiality, an element of step three, was generally not a concern, partly because anyone who overhears the phone conversation overhears only one side of it, but this was also the result of considerate providers who first asked about the patient's availability. As one woman noted, “…it hasn't been hard for me because when the health provider calls, he first asks me if I am in a position to have a conversation with him…” *39 y.o. female, Uganda (OPD)*.

Providers’ considerateness during these phone visits, and their frequency, built clients’ confidence and facilitated their openness to sharing problems. The availability of phone visits also reassured clients that they had someone who could advise them about challenges as they arose.

***Patient‐friendly care***. Regardless of location, attentive and friendly providers made clients feel respected. Clients trusted in the knowledge and competence of providers and felt providers had their wellbeing in mind.

“…the provider is very friendly – you know it is very difficult to go for PrEP in the shop – and the fact that I can come and share with the provider here is something that is important to me… I liked the way the provider was talking politely and in a friendly way…” *19 y.o. male, Kenya (OPD)*

In this way, patient‐friendly care opened up social space, supporting step three (addressing constraints to uptake) as well as step four (supporting ongoing usage of medications).

#### 3.1.3 Method switching and choice

Most narratives about method switching suggest clients appreciated feeling in control of their choices; a minority still felt unable to imagine making their own choices and deferred to providers’ opinions.

***Learning about prevention options***. Participants were happy to learn about available HIV prevention options, and often were surprised to learn multiple methods existed. These discussions sometimes served as a catalyst to steps one and two. Younger participants noted how useful these options will be when extended to the larger community:

“…When I was informed about the study, I was impressed that it can help the general population, especially the youth. This is because youths are at risk of contracting various diseases – not only HIV. I therefore thought it could help us a lot. I have many friends who are suffering, yet there are no solutions to their problems…If they can learn about them and take up the prevention services, then they can take care of their lives…” *18 y.o. male, Kenya (OPD)*

***Providers’ instructions***. Providers instructed participants on how to use each method, reassured patients about their safety and allowed participants to choose which methods would be most appropriate for them. A CHW described the questions participants asked, and the benefits of being present to answer them:

“…They would ask if there were pills to take if they had been exposed to HIV to avoid the risk of getting infected, and those who were already infected how not to spread the virus or get re‐infected… I was very happy about it because it helps one protect them from acquiring HIV and also if already exposed, it helps one from spreading [HIV]…” *40 y.o. female, Uganda (CHW)*

In several instances, provider guidance was a concomitant essential in helping promote the uptake of prevention, but only in the context of offering a choice of methods they could select, refuse or change if they felt like it. While this is a component of the mechanism of the intervention, channelling the flow of actions, it is not necessarily a step in itself on any particular pathway.

***Option to switch products***. As participants became more aware of prevention options, their risk and their experiences with methods, they began to opt for the “best fit” according to what was happening in their lives at the time:

“…For me both PEP and PrEP are good. At first, I chose PEP as an option, then later switched to PrEP. I picked PEP because I did not want to take the drugs daily at the time, because I did not have any permanent sexual partner… I switched because I knew that at some point, I would get a partner and we may not find time to come and see the provider.” *22 y.o. female, Kenya (OPD)*

In part, participants gained the confidence to make these decisions because of the counselling the providers gave them on the different options, including the potential introduction of injectable PrEP, and the option of switching to it in the future: “…if I decided on an injection, my decision would be made based on an informed decision after being taught about the different methods by the doctor…” *51 y.o. female, Uganda (OPD)*

Support and encouragement at each step in the process seemed to lead to greater client self‐efficacy for adherence to prevention.

### 3.2 Countervailing Forces

Several factors impede prevention method adoption, despite positive experiences with DCP delivery. These include limited community knowledge about prevention options, persistent stigma, lack of partner or familial support, infrastructure challenges around phone service and concerns about medications. As shown in Figure 1, each inhibits a particular step in the pathways: lack of knowledge applies to steps one and two, stigma and lack of family support are social constraints implicated in step three and concerns about medication potentially relate to steps one, four and five.

***Community contexts of stigma***. Pervasive stigma had varied impacts on individuals. Some feared being seen by others during clinic; others worried about rumours if visited at home. Participants reported that they and other DCP clients were well‐informed about prevention options, but other community members were not, especially regarding the distinction between PrEP and ART. However, community perceptions changed as people became more familiar with the study:

“…Initially, people did not like anything to do with PrEP because of the stigma attached to it; if I take PrEP someone would think that I am on ARVs or I want to be promiscuous. Nowadays, even the ladies we handle are comfortable with sending their men to come for their refills; meaning they have understood that PrEP is not ARV but something that prevents HIV …” *46 y.o. female, Kenya*

Even so, the idea that people take PrEP in order to permit promiscuity without repercussions remained for some. One male youth even felt the burden of daily oral PrEP might help prevent “immorality” compared to injectable PrEP:

“…It [injectable PrEP] is good and bad at the same time; it is bad because the level of sexual immorality will be on the rise and most youths would be engaged so much, unlike when you are taking the pills, you would have some moderation….” *19 y.o. male, Kenya (OPD)*

***Family relationships***. Discussing the use of prevention methods within intimate partnerships was sometimes challenging for couples. Although some partners were open with one another about their PrEP use, this was not universal; several participants described having discontinued PrEP due to a lack of partner support. In the following instance, a husband overcame his wife's initial opposition:

“…Now regarding taking a daily pill, for us with wives, when your wife sees you taking the pill, she will think that you are HIV positive, and you see with our village wives, it may cause a problem at home. She asked me why I had brought HIV drugs at home. I explained to her that the drugs are meant to help someone from acquiring HIV… I later requested her to come along with me to the health facility so that she is educated more about the preventive drugs. She agreed and [we] tested for HIV together… we protect ourselves from HIV very well…” *19 y.o. male, Uganda (OPD)*

Several participants expressed concern that using a prevention method within a relationship could signal a lack of trust, eliciting blame or worse. In‐laws and relatives often joined conversations about PrEP use, enacting stigma via shaming, since it raised concerns about the family's image. As one woman reported,

“…I thought someone may find out and tell my husband and other people… my husband is not an easy man and [this] place has a lot of gossip… I call the nurses and at times they come… they came with boda‐boda and at times they hid. My father–in–law tried to inquire what they wanted but they said they assisted in the delivery of the baby I had, and had come for polio [vaccination].” *20 y.o. female, Uganda (ANC)*

Although home visits were helpful, providers sometimes had to have a “cover story” for their purpose in delivering HIV medication at home. Overall, husbands and mothers‐in‐law were most often mentioned as those who may forbid women from taking PrEP. Yet, both women and men felt that injectable PrEP might smooth over domestic relations when it comes to using a prevention option.

“…I would go for the injection because once taken, one is assured that they are protected and [. . .] the partner will not know since they are not going to check or test your body. But pills can easily be seen…” *33 y.o. female, Uganda (ANC)*

“…It also works because it eliminates those issues of disclosure among partners because if one is taking the injection without the partner's knowing, then it remains so. If she gets to see tablets in your pockets then that starts quarrels. An injection is given in privacy and no one needs to know…” *32 y.o. male, Uganda (OPD)*

Many people reported that men's fear of the needle prick required for blood tests was an impediment to couple's testing. In addition, the social permissibility of men to be promiscuous was felt to have its downsides, and men sometimes sought to conceal their behaviour from their female partners and avoid testing as a couple:

“…Most of the time we go as an individual and in secret because I am afraid, depending [on] how I have mingled with other women. I fear that the result might not be that good. I need to go and check it as an individual first. However, partner testing is very good because you test when you are together…” *37 y.o. male, Kenya (VHT)*

Another challenge participants expressed around HIV self‐tests was the absence of a counsellor. Some participants cautioned that testing by oneself is not for everyone:

“…I would say that the one being done by the provider is much better than self‐testing, because the provider will first take you through counseling unlike when you do it by yourself and the result turns out to be positive. You can end up committing suicide…” *19 y.o. male, Kenya (OPD)*

Participants expressed caution with self‐testing, should the test turn out positive. Some wanted the assurance of having a provider within reach, to help them navigate a change in serostatus and link them to care. Others worried the self‐test could be more discoverable by family or community members, whereas testing at facilities was relatively confidential. On balance, participants favoured the self‐testing option simply for the rapid confirmation of serostatus.

***Infrastructure barriers (phone service)***. Despite positive reports around phone reminders, participants living on islands and remote areas had spotty or unavailable cell phone service. Although some participants with poor network connectivity were able to find a work‐around via other technologies or hotspots, they found it easier simply to go to facilities for care.

***Properties of the medications***. For some participants, misunderstandings led to doubts about the efficacy of prevention products. Because PrEP looks identical to earlier ART, participants worried they would be assumed to be living with HIV if seen with PrEP. Some clients even believed the doctor prescribed them ART because they were actually living with HIV and the doctor was deceiving them to spare their feelings. A few participants remarked that the pills were large, difficult to swallow and smelly. Others mentioned pill burden. Coming for refills was also challenging. In contrast, participants anticipated that long‐acting injectable PrEP would ameliorate many burdens, including the problem of forgetfulness that interfered with daily pill routines:

“…The injection would be good because with the pills, one can forget to take them, but the injection keeps within the blood [. . .] you spend a long time before taking another one. But the pills are taken every day, so if you get busy with work or on a journey, you might just go to sleep without taking [them]…” *41 y.o. female, Uganda (OPD)*

Thus, participants identified injectable PrEP as a single solution to the problems of pill size, burden, risk of unwanted disclosure and forgetfulness that challenged adherence.

## 4 DISCUSSION

This qualitative study showed pathways through which a structured, patient‐centred HIV prevention delivery model in Kenyan and Ugandan communities worked to facilitate clients’ uptake of HIV prevention. The results indicate that the DCP intervention fostered a positive feedback loop, beginning with health literacy, supporting self‐efficacy and justification to others, and continuing through persuading others of the benefits of prevention. Each step potentially altered the local context, opening more social space for participants to choose prevention methods and to reassess their choices over time. Intervention elements and countervailing factors acted to help individuals jump over certain steps or impede their advancement. The contexts in which people decided to take up or switch options were thereby similarly altered, as disclosures of HIV status and PEP/PrEP use, and opportunities for “vicarious efficacy” for self‐testing and PEP/PrEP use (confidence in one's ability to take up a new behaviour after seeing similar others succeed in doing so) [23] accumulated in the communities over time. Findings provide evidence that offering a patient‐centred care model that includes offering a choice of HIV prevention options, locations and delivery modalities, and flexible, competent, and friendly care provision, is one where clients feel valued and empowered to prioritize their health.

Several studies indicate people embrace choice among HIV prevention options [10, 28]; our study suggests that a patient‐centred approach undergirds optimal prevention delivery: clients must first be introduced to the range of options and then instructed on how to use them. When given by providers attentive to the constraints and opportunities afforded by clients’ circumstances, such instruction engenders patients’ trust. Providers must also trust clients and be open to answering their questions, encouraging their decisions and accepting when they want to switch methods (or discontinue) as circumstances change. Clients must have the opportunity to try methods and learn what works for them; such experimentation engenders self‐efficacy for method usage and confidence in the method's effectiveness. Together with the above pathway(s), these components form a self‐reinforcing loop which increases health literacy, a sense of self‐efficacy, and, depending on the patient's social position, an ability to successfully justify one's actions to others—and at times also to persuade others to adopt a prevention method.

Findings highlight that even with interventions designed to optimize opportunities to engage in HIV prevention, contextual factors can countervail their effectiveness. Home visits particularly highlight how the intervention's access and choice approach facilitated uptake, yet social barriers potentially undercut those efforts. Several factors unrelated to implementation also hindered uptake, across the broad categories of community beliefs, norms and stigma; limited literacy (experience/knowledge) around prevention methods; highly gendered power dynamics within intimate partnerships and families; and adverse experiences due to the properties of the products themselves.

Our team [29] and others [30, 31, 32, 33] have previously reported how pervasive HIV‐related stigma influences considerations around HIV prevention despite indications that stigma is declining through clearly marked pathways [34, 35]. Until community discussions about new prevention methods reach a critical threshold, stigma may remain prominent [36, 37, 38]. The advent of PrEP reignited HIV stigma and debates about sexual morality in study communities [39, 40]. Here, we highlight *how* stigma influences prevention, as the impact of stigma on prevention uptake manifested differently for different individuals whether fear of being seen at the local clinic or during home visits, or how partners and in‐laws might react. People perceived self‐testing as a boon for avoiding the stigma associated with facility‐based testing. For others, this benefits their desire for support from a provider at the moment they obtained a result that outweighed potential stigma. The similarity of PrEP to ART led to concerns that those who use PrEP (or PEP) would be mistakenly assumed to be living with HIV.

Population‐level oral PrEP implementation is still recent vis‐à‐vis its social diffusion [41], and participants felt PrEP to be “new” and for many, unfamiliar. Incomplete knowledge can hinder the proper use of PrEP [42]. Participants overall had a good knowledge of PrEP, but often confused PEP and PrEP, which is common when people are introduced to the methods [43, 44, 45]. Notably, participants eagerly anticipated the long‐acting injectable PrEP, since its attributes—especially clandestine use—promise to circumvent several barriers.

### 4.1 Limitations

Interviews were conducted at one time point; a longitudinal design could have strengthened the evidence for the proposed pathways of action. As a qualitative study conducted in rural communities in Uganda and Kenya, findings reflect the specific social and cultural contexts of those settings. Our design is strengthened by its large (for qualitative research) and strategically composed sample, and the full participation of interviewers from the settings in the data interpretation.

The potential sustainability of the DCP model was enhanced by its leveraging of the CHW workforce, a sponsored programme of Kenya's and Uganda's Ministries of Health. For the trials, CHWs received ongoing training on HIV prevention and dynamic choice, and all medical decisions were made by facility‐based healthcare providers in conjunction with clients. Support for government expansion of CHW roles is increasing, with the recognition that healthcare delivery needs to reach outside brick‐and‐mortar facilities, although this may not be replicable in the short term. Research to measure the cost‐effectiveness of the model is needed and ongoing. Task‐shifting also may require higher‐level policy decisions.

## 5 CONCLUSIONS

The diverse challenges to prevention uptake faced by individuals in rural community settings in Kenya and Uganda means no “one size fits all” solution is available; rather, the flexibility of choice in this patient‐centred model may have helped increase its reach to a wider set of persons at risk of HIV—beyond facility‐based prevention offered at HIV clinics—expanding the set of opportunities available to individuals to engage in HIV testing and choose a prevention method.

## AUTHORS’ CONTRIBUTIONS

CSC, TA and JJ‐P led the analysis and wrote the manuscript, with contributions to analysis from JA, CA, FA, AO and LO. JA, FM, LO, FA, CA and AO conducted data collection, and along with JJ‐P conducted curation and coding of the transcripts. MRK, MLP, GC, EK, LBB, JA and DVH conceived the parent study, provided field‐based oversight and revised critically. CSC conceived the qualitative project. All authors have read and approved the final version.

## COMPETING INTERESTS

None of the authors have any financial or non‐financial interests that are directly or indirectly related to the work submitted for publication.

## FUNDING

Research reported in this manuscript was supported by the U.S. National Institute of Allergy and Infectious Diseases (NIAID), the National Heart, Lung, and Blood Institute (NHLBI), and the National Institute of Mental Health (NIMH) and co‐funded under award number U01AI150510 (Havlir).

## DISCLAIMER

The content is solely the responsibility of the authors and does not necessarily represent the official views of the NIH.

## Data Availability

De‐identified study data and a data dictionary will be made available in a secure online repository following SEARCH Scientific Review Committee approval of a concept sheet summarizing the analyses to be done, with a signed data access agreement. Further inquiries can be directed to the SEARCH Scientific Review Committee via Carol.Camlin@ucsf.edu.
